# Vildagliptin Can Alleviate Endoplasmic Reticulum Stress in the Liver Induced by a High Fat Diet

**DOI:** 10.1155/2018/5045182

**Published:** 2018-03-12

**Authors:** Xiaoqing Ma, Wenhua Du, Shanshan Shao, Chunxiao Yu, Lingyan Zhou, Fei Jing

**Affiliations:** ^1^Department of Endocrinology, Shandong Provincial Hospital Affiliated to Shandong University, Jinan, Shandong 250021, China; ^2^Shandong Provincial Key Laboratory of Endocrinology and Lipid Metabolism, Jinan, Shandong 250021, China; ^3^Institute of Endocrinology and Metabolism, Shandong Academy of Clinical Medicine, Jinan, Shandong 250021, China; ^4^Department of Endocrinology, Jining No. 1 People's Hospital, No. 6 Health Road, Rencheng, Jining 272011, China; ^5^Department of Endocrinology, Linyi People's Hospital, Linyi, Shandong 276001, China; ^6^Department of Endocrinology, The Second Hospital of Shandong University, No. 247 Beiyuan Street, Jinan 250033, China

## Abstract

*Purpose. *We investigated whether a DDP-4 inhibitor, vildagliptin, alleviated ER stress induced by a high fat diet and improved hepatic lipid deposition.* Methods. *C57BL/6 mice received standard chow diet (CD), high fat diet (HFD), and HFD administered with vildagliptin (50 mg/Kg) (V-HFD). After administration for 12 weeks, serum alanine aminotransferase, glucose, cholesterol, triglyceride, and insulin levels were analyzed. Samples of liver underwent histological examination and transmission electron microscopy, real-time PCR for gene expression levels, and western blots for protein expression levels. ER stress was induced in HepG2 cells with palmitic acid and the effects of vildagliptin were investigated.* Results.* HFD mice showed increased liver weight/body weight (20.27%) and liver triglycerides (314.75%) compared to CD mice, but these decreased by 9.27% and 21.83%, respectively, in V-HFD mice. In the liver, HFD induced the expression of ER stress indicators significantly, which were obviously decreased by vildagliptin. In vitro, the expressions of molecular indicators of ER stress were reduced in HepG2 when vildagliptin was administered.* Conclusions.* Vildagliptin alleviates hepatic ER stress in a mouse high fat diet model. In HepG2 cells, vildagliptin directly reduced ER stress. Therefore, vildagliptin may be a potential agent for nonalcoholic fatty liver disease.

## 1. Introduction

DPP-4 (also known as CD26) was first described in 1966 by Hopsu-Havu and Glenner by its enzymatic activity in rat livers; it is a membrane-associated peptidase, which is expressed ubiquitously on the apical surfaces of epithelial and acinar cells, in endothelial cells, fibroblasts, and lymphocytes, and also as a soluble circulating form in the plasma [1]. DPP-4 expression is influenced by hypoxia-inducible factor-1*α* (HIF-1*α*), hepatocyte nuclear factor-1*α* (HNF-1*α*), interferons, retinoic acid, and various cytokines. Glucagon-like peptide-1 (GLP-1) is a representative target of DDP-4, which degrades GLP-1 into an inactive substance. GLP-1 is an incretin, which is glucose-dependent and is released into the blood, inhibits glucagon secretion, promotes insulin secretion, and reduces blood glucose levels in patients with type 2 diabetes. In recent years, studies have shown that GLP-1 receptor agonist can promote weight loss, protect vascular endothelial cells, reduce a variety of cardiovascular risk factors, and potentially play an anti-inflammatory role. In 2006, a DDP-4 inhibitor was approved for the treatment of type 2 diabetes mellitus because it prevents the hormone GLP-1 from degradation [2, 3]. In recent years, growing clinical and animal experiments suggest that DDP-4 inhibitors could have many nonglycemic actions. DPP-4 inhibitors may reduce myocardial infarction damage [4], ameliorate myocardial ischemia-reperfusion injury [5], assist vasoconstriction [6], and suppress atherosclerosis [7]. Recent studies showed that DDP-4 inhibitors—linagliptin, sitagliptin, and MK-0626—can all improve hepatic steatosis [8–11]. A clinical study also showed that vildagliptin can decrease liver triglyceride content by 27% in type 2 diabetes mellitus patients compared to placebo [12], but the specific mechanism is still not clear.

The endoplasmic reticulum (ER) is a dynamic organelle in eukaryotic cells that participates in the synthesis of proteins, calcium homeostasis, phospholipid synthesis, and lipid metabolism [13]. Under stressful situations, protein maturation in the ER is impaired, and then misfolded proteins accumulate in the ER lumen, which leads to a characteristic stress response named the unfolded protein response (UPR), which is mediated by activating three protein sensors: protein kinase RNA- (PKR-) like ER kinase (PERK), inositol-requiring enzyme-1 (IRE1), and activating transcription factor 6 (ATF6). However, prolonged UPR activation is related to many metabolic diseases, such as insulin resistance, type 2 diabetes, and hepatic steatosis [14–16]. Studies show that inhibition of ER stress leads to a decrease in hepatic steatosis [17, 18].

Based on this background, we hypothesized that inhibition of DDP-4 may reduce ER stress. Therefore, the purpose of this study was to investigate the action of the DDP-4 inhibitor, vildagliptin, on ER stress induced by high fat diet in mice and observe the resulting effect on hepatic steatosis; simultaneously, we aimed to investigate the direct action of vildagliptin on ER stress in HepG2, a human cell line.

## 2. Materials and Methods

### 2.1. Animals

**Figure 1:**
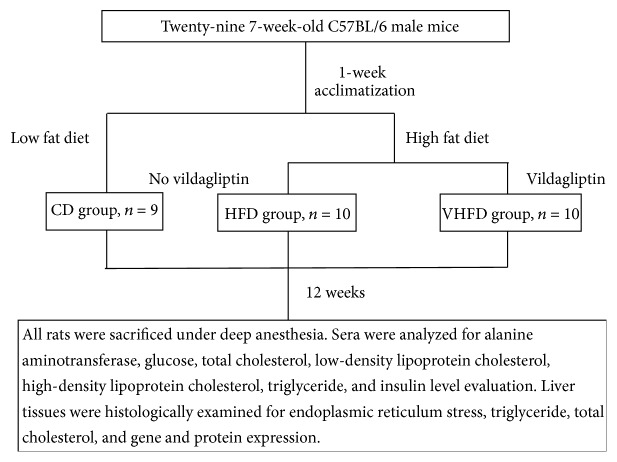
Flowchart of the experiment.

Twenty-nine seven-week-old C57BL/6 male mice were purchased from Vital River Corporation (Beijing, China) and were housed in an air-conditioned room (temperature of 20 ± 2°C, relative humidity of 60 ± 10%) in a 12-hour light/dark cycle environment, with free access to food and water. All mice were allowed to acclimatize for 7 days before the experiments. The mice were then randomly divided into three groups: CD group (*n* = 9) received a standard chow diet (14.09 KJ/g) (CD + vehicle), HFD group (*n* = 10) received a simple high fat diet (17.05 KJ/g) (HFD + vehicle), and V-HFD group (*n* = 10) received a high fat diet supplemented with vildagliptin (HFD + vildagliptin) (Figure 1). The simple HFD contained 82.7% CD supplemented with 2% cholesterol, 15% lard (31.29% KJ), and 0.3% sodium cholate. There was no significant difference in weight between the three groups before treatment. Vildagliptin (50 mg/Kg per day) [19] was given by intragastric administration for 12 weeks from 7 weeks old to 19 weeks old in the V-HFD group; meanwhile, distilled water (0.1 ml per 10 g weight) was given by intragastric administration to the CD and HFD group mice. The composition of the experimental diets was assayed by Beijing Research Institute for Nutritional Resources, and details are shown in Table 1. The body weights of the animals were monitored weekly for the duration of the study.

This study was carried out following the recommendations from the* Guide for the Care and Use of Laboratory Animals* of the National Institutes of Health. The protocol was approved by the Shandong University Institutional Animal Care and Use Committee (Jinan, China). All efforts were made to minimize suffering for the animals.

### 2.2. Serum Biochemical Assays

All mice were euthanized using pentobarbital sodium after being fasted for 8 h; fasting 1 ml blood samples were obtained by subclavian venous puncture. Serum was obtained by centrifugation of blood at 3000 rpm for 15 min. Alanine aminotransferase (ALT) was measured as an indication of liver function by an automated hematology analyzer (Bayer, PA, USA). Serum glucose and lipid parameters including total cholesterol (TC), low-density lipoprotein cholesterol (LDL-C), high-density lipoprotein cholesterol (HDL-C), and triglyceride (TG) were measured using Olympus AU5400 automatic analyzer in Shandong Provincial Hospital. Serum insulin levels were tested by mouse insulin ELISA Kit (CSB-E05071m).

### 2.3. Coefficient of Liver to Body Weight and Liver Histopathology

Mice were weighed before overdose anesthesia sacrifice, their livers were removed and weighed, and one part was immediately fixed in 4% paraformaldehyde for hematoxylin and eosin staining, while another part was frozen in liquid nitrogen for Oil Red O staining to observe the histopathological changes in the liver tissue using a microimaging system (Axio Imager A2, Zeiss, Jena, Germany). The coefficient of liver to body weight was calculated as the ratio of liver wet weight (g) to body weight (g).

### 2.4. ER Structure

For transmission electron microscopic observation, the liver tissues were fixed in buffered 3% glutaraldehyde and 1% osmium tetroxide, dehydrated through an ethanol series, and embedded with epoxy resin. Ninety-nanometer sections were cut using an LKB-V ultramicrotome (LKB, Bromma, Sweden) and doubly stained with uranyl acetate and lead citrate. The ER ultrastructure was observed and photographed using a transmission electron microscope (H-800, Hitachi, Tokyo, Japan).

### 2.5. Hepatic Triglyceride and Total Cholesterol Assays

The triglyceride content and total cholesterol in the liver were assayed using a triglyceride assay kit (E1015; Applygen Technologies, Beijing, China) and a total cholesterol assay kit (GPO-POD; Applygen Technologies, Beijing, China), respectively, according to the manufacturer's recommended protocol.

### 2.6. RNA Extraction and Real-Time PCR

Total RNA was extracted from mouse liver tissue using TRIzol (TAKARA Bio Inc., Japan); 5 ug RNA was then reverse-transcribed using random hexamer primers (TAKARA). Real-time PCR was performed with SYBR Premix Ex Taq II (TAKARA) in a final volume of 20 ul including 250 ng of reverse-transcribed total RNA, 10 ul of 2x SYBR Premix Ex Taq, and 400 nM of primers. The primers used are listed in Table 2. PCR was carried out for 40 cycles of 95°C for 15 sec, 60°C for 30 sec, and 72°C for 30 sec in a LightCycler instrument (Roche Diagnostics). Gene expression was determined by relative quantification by the 2^−ΔΔCT^ method.

### 2.7. Western Blot Analysis

Liver tissue samples were homogenized in total protein solution and separated by centrifugation after lysis was considered to be sufficient. The supernatant was collected and the protein concentration was measured using the BCA method. The proteins (90 ug) were then separated using sodium dodecyl sulfate polyacrylamide gel electrophoresis (SDS-PAGE) with 10% polyacrylamide gels and transferred to polyvinylidene fluoride (PVDF) membranes (Millipore, Billerica, MA, USA). The membranes were incubated with primary antibodies at 4°C after being blocked (0.5% nonfat milk powder in TBST) for 1 h. The primary antibodies used were for the following proteins: binding immunoglobulin protein (BiP) (1 : 1000), PKR-like ER kinase (PERK) (1 : 500), phosphor-PERK (sc-32557) (1 : 200), eukaryotic initiation factor 2 (eIF2*α*) (1 : 1000), phospho-eIF2*α* (1 : 1000), inositol-requiring enzyme-1 (IRE1) (1 : 1000), phospho-IRE1 (1 : 800), X-box binding protein-1 (xBP-1) (1 : 200), and *β*-actin (1 : 3500). And they were all from Cell Signaling Technology (Boston, USA). The immune complexes were detected after incubation with either anti-rabbit or anti-mouse secondary antibodies. To estimate the protein expression levels, the relative target protein levels were normalized to *β*-actin in the same membrane.

### 2.8. Immunohistochemistry

Sections were handled by standard techniques, and heat treatment to retrieve the antigen sites was performed. The sections were treated with 3% hydrogen peroxide in dH_2_O at room temperature to quench endogenous peroxidases and incubated for 1 h at room temperature with blocking solution and then with primary antibodies overnight at 4°C. The primary antibody was rabbit polyclonal antibody against BiP (1 : 75). For the negative control, IgG was added instead of the primary antibody. The reactivity of the antibodies was detected using a PV-6001 polink-1 HRP DAB detection system, one-step polymer detection system for mouse. The nuclei were counterstained by hematoxylin. Positive staining appeared as a brown-yellow color.

### 2.9. Cell Culture

The HepG2 human cell line was purchased from the Chinese Academy of Science (Shanghai) and was cultured in Eagle's minimum essential medium (EMEM) (Gibco) containing 10% fetal bovine serum and 100 U/ml penicillin-streptomycin in a humidified incubator with 5% CO_2_ at 37°C. The cells were exposed to different concentrations of palmitic acid (PA) or 1 *μ*M thapsigargin as previously described [20–24]. The effect of vildagliptin was investigated by its addition at various concentrations after the cells reached 80%–90% confluence.

### 2.10. Statistics

Data were analyzed using SPSS 22.0 (IBM, USA) and are expressed as the mean ± standard deviation. Differences between means were compared using either an unpaired Student's *t*-test for two-group comparisons or one-way analysis of variance (ANOVA) (Dunnett's *t*-test or LSD test) for multiple comparisons. Differences were considered significant at *P* < 0.05.

## 3. Results

### 3.1. Blood Biochemistry

Blood glucose of mice in the HFD group increased compared to the CD group (*P* < 0.001, Table 3), but it decreased in the V-HFD group compared to the HFD group (*P* = 0.038, Table 3). Similarly, insulin was the highest in the HFD group (*P* = 0.031 compared with the CD group, Table 3) and decreased in the V-HFD group (*P* = 0.042 compared with the HFD group, Table 3), but it was the lowest in the CD group. Mice in the HFD group had lower triglyceride levels compared to those in the CD group, but the differences were not significant; triglyceride levels were significantly increased in V-HFD mice (*P* = 0.013, Table 3). Total cholesterol levels in the HFD group mice were higher than in the CD group mice (*P* < 0.001, Table 3), but total cholesterol was not significantly changed in the V-HFD group compared to the HFD group. Compared to the CD group, LDL cholesterol showed higher levels in the HFD group (*P* < 0.001, Table 3) and then decreased in the V-HFD group (*P* = 0.034, Table 3). Interestingly, compared with the CD group, the HDL cholesterol increased in the HFD group (*P* < 0.001, Table 3) but was not changed by vildagliptin treatment. ALT levels increased significantly in the HFD group (*P* = 0.006, Table 3) and decreased obviously in the V-HFD group (*P* = 0.03, Table 3).

### 3.2. HFD Mice Supplemented with Vildagliptin Showed Moderate Intrahepatic Lipid Deposition

**Figure 2:**
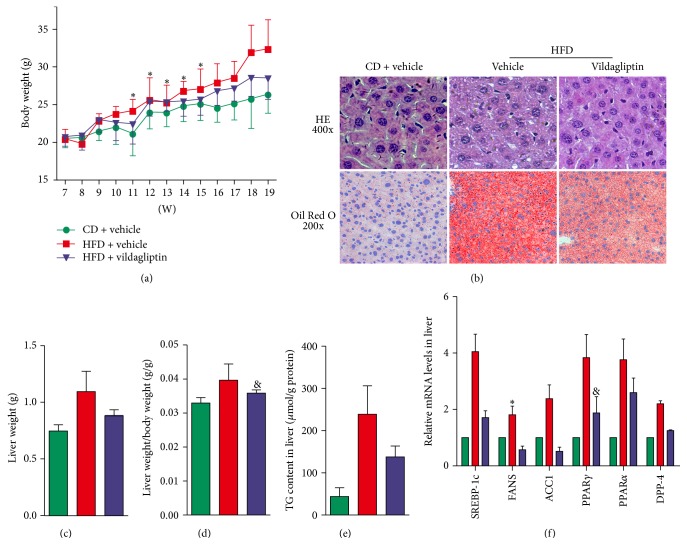
Vildagliptin reduced hepatic lipid deposition. (a) Weights of the mice in the three groups during the experiment (*n* = 9 per group). (b) Liver tissue stained with H&E (upper panel, magnification ×400); liver tissue sections stained with Oil Red O (lower panel, magnification ×200). (c) Liver wet weights were measured (*n* = 7–9). (d) The liver index was calculated using liver wet weight/body weight (*n* = 7–9). (e) The triglyceride (TG) content in the liver was measured and normalized with the protein content (*n* = 7–9). (f) Relative mRNA levels of genes related to the synthesis of TG and dipeptidyl peptidase-4 (DDP-4). Expression values were normalized to *β*-actin mRNA. Data was presented as the means ± SD; ^*∗*^*P* < 0.05 relative to the CD group; ^&^*P* < 0.05 relative to the HFD group.

Body weight increased in all groups over the study period as the mice grew, but the growth rates were higher in the HFD group and lower in the V-HFD but the lowest in the CD group (Figure 2(a)). In ER stress states, hepatic lipogenesis occurs via the SREBP-1c pathway [20]. Oil Red O staining of liver tissue sections showed slight hepatic steatosis in V-HFD group mice and higher levels of steatosis in the HFD group mice (Figure 2(b)). The liver weight and liver weight/body weight were both the highest in the HFD group. Mice in the V-HFD group showed slightly higher liver weight and liver weight/body weight than the CD group (Figures 2(c) and 2(d)); the liver weight/body weight decreased by 9.26% compared to HFD mice. Triglyceride content was significantly increased in the liver of HFD mice by 443.41% (Figure 2(e)) compared to CD mice; in the liver of V-HFD mice, triglyceride content decreased significantly by 42.42% compared to HFD mice. mRNA expressions of genes associated with lipid synthesis, SREBP-1c, FAS, and ACC1 were all upregulated in HFD mice and downregulated in V-HFD mice. PPAR*α* and PPAR*γ* were related to lipid metabolism. In HFD mice, mRNA expressions of PPAR*α* and PPAR*γ* were both increased, but they decreased in V-HFD mice (Figure 2(f)).

### 3.3. Vildagliptin Can Ameliorate High Fat Diet Induced ER Stress in the Liver

**Figure 3:**
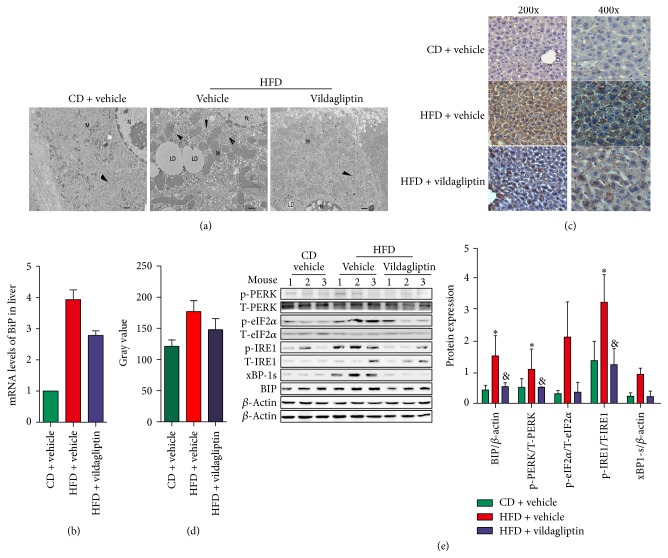
Vildagliptin ameliorated high fat diet induced endoplasmic reticulum (ER) stress. (a) Electron microscope (magnification ×15000) analyses of the ER in livers of mice from CD, HFD, and V-HFD groups. Scale bars represent 2 *μ*m. (b) The mRNA levels of BiP measured by RT-PCR; data were normalized according to *β*-actin levels. (*n* = 5 per group). (c) The expression of BiP was assessed using immunohistochemical staining (magnification ×200 and magnification ×400). (d) The semiquantitative analysis of staining intensity was conducted using ImageJ software. (e) ER stress associated markers BiP, p-PERK, p-IRE1*α*, p-eIF2*α*, and xBP-1s expression in the livers of mice investigated by western blotting. Each expression level was quantified by densitometry, normalized with *β*-actin, and the relative phosphorylated protein levels were normalized with the corresponding total protein level. Data was represented as the means ± SD; ^*∗*^*P* < 0.05 relative to CD group; ^&^*P* < 0.05 relative to HFD group.

Electron microscopy showed lipid droplets in the hepatocytes of HFD mice, ER dilatation, disordered ER, and a decreased number of particles (Figure 3(a)); however, each of these indications of ER stress was significantly reduced after using vildagliptin, or even disappeared (Figure 3(a)). The ER stress marker BiP decreased in both mRNA and protein levels with the application of vildagliptin (Figures 3(b), 3(c), and 3(d)); similarly, the expression of ER stress markers, such as p-eIF2a, p-IRE1a, and xBP-1, was also reduced in V-HFD mice (Figure 3(e)).

### 3.4. Vildagliptin Ameliorated ER Stress In Vitro

**Figure 4:**
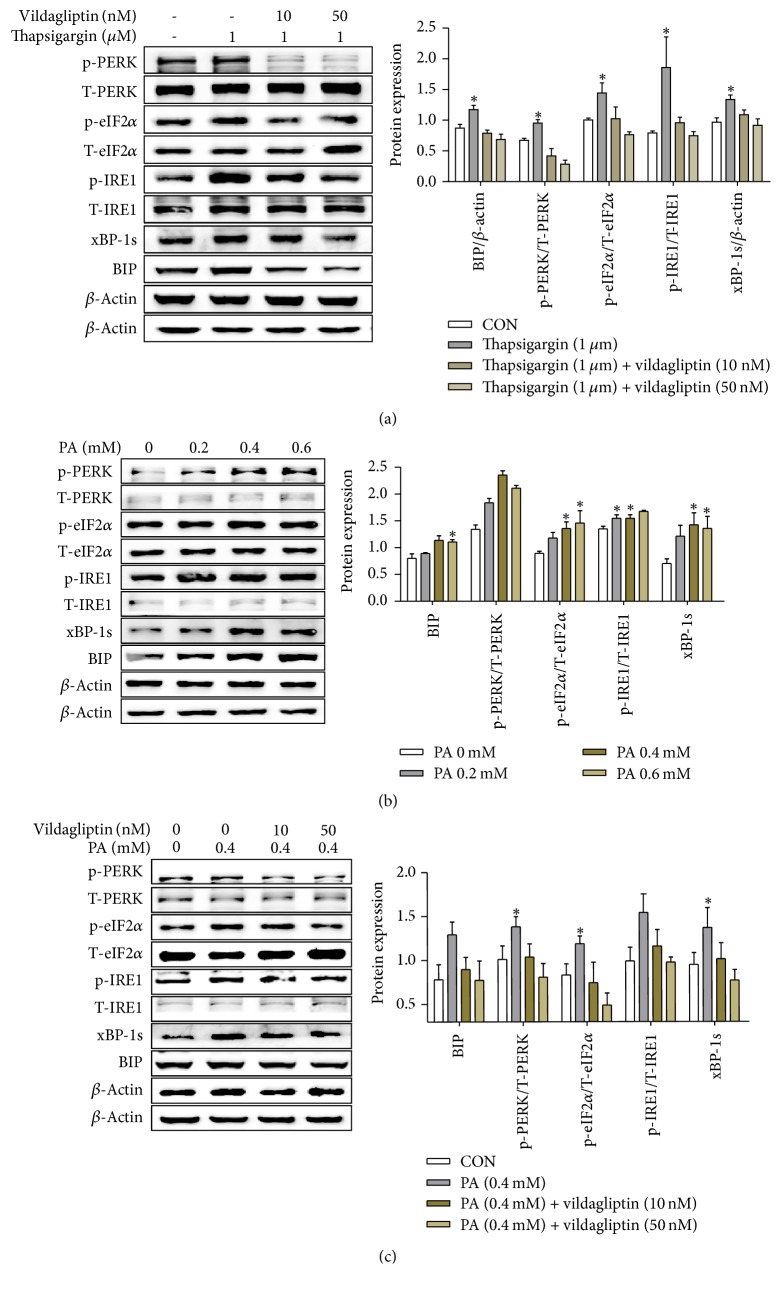
Vildagliptin ameliorated ER stress induced by PA or thapsigargin in HepG2 cells. (a, c) HepG2 cells were treated with 1 umol/L thapsigargin or 0.4 mmol/L PA with the indicated concentrations of vildagliptin for 24 h. Proteins were analyzed by western blotting using anti-BiP, p-PERK, p-eIF2*α*, p-IRE1*α*, and xBP-1 antibodies and quantified by densitometry (*n* = 3 for each condition). (b) HepG2 cells were treated with different concentrations of PA (in DMEM) for 24 h after the cells were starved for 3 h. Proteins were analyzed by western blotting using p-PERK, p-eIF2*α*, p-IRE1*α*, xBP-1, and BiP antibodies and quantified by densitometry (*n* = 3 for each condition). Data was represented as the means ± SD; ^*∗*^*P* < 0.05 relative to PA (0 mM).

PA concentration-dependently increased levels of ER stress molecular markers, such as p-PERK and p-eIF2*α* in HepG2 cells (Figure 4(b)). Then, cells were treated with 0.4 mM PA with or without vildagliptin; we observed that the levels of p-eIF2*α*, p-IRE1*α*, and xBP-1 all increased after PA addition but decreased when dealing with vildagliptin (Figure 4(c)). In order to confirm the direct action of vildagliptin ameliorating ER stress in vitro, HepG2 cells were also treated with thapsigargin and various concentrations of vildagliptin to induce ER stress. The result revealed that thapsigargin induced ER stress was alleviated by vildagliptin (Figure 4(a)).

## 4. Discussion

In this study, we investigated the action of the DDP-4 inhibitor, vildagliptin, on ER stress and triglyceride content in the livers of mice fed with a high fat diet. Consistent with the literature, ER stress in the liver was induced successfully by a high fat diet in our study, but we then found that ER stress was alleviated to a certain extent by treatment with vildagliptin. V-HFD mice showed lower increases of liver weight/body weight and liver triglycerides than HFD mice. The HFD induced expression of ER stress indicators, such as BiP, p-PERK, p-eIF2*α*, p-IRE1, and xBP-1, was also inhibited by vildagliptin. These results suggest that in the future vildagliptin may be useful as a treatment for nonalcoholic fatty liver disease (NAFLD).

Some previous studies have also suggested that DDP-4 inhibitors may have a role to play in preventing ER stress. Studies have shown that linagliptin may be able to protect patients with diabetic cardiomyopathy from IRE1*α*-XBP-1 pathway-mediated ER stress [21, 22]. And linagliptin also protected pancreatic INS-1 cells from PERK and IRE1 pathway-mediated ER stress induced by thapsigargin [23]. Sitagliptin can attenuate steatohepatitis by suppressing the ER stress in the liver induced by a methionine/choline-deficient (MCD) diet in C57BL/6 mice [24]. Vildagliptin also can both increase *β*-cell mass and preserve the functions of pancreatic *β*-cells by suppressing ER stress and oxidative stress in a mouse model of diabetes [19]. In our study, we found that large lipid droplets were visible in the liver cells, the ER was irregular and was enlarged, and the particles on the ER were reduced in the HFD mice compared to CD mice, but when the mice were treated with vildagliptin, these phenomena were obviously alleviated and returned towards normal ER structures. We further demonstrated that vildagliptin also attenuated the high fat diet induced liver ER stress by reducing PERK-eIF2*α* and IRE1*α*-XBP-1 phosphorylation. So, for the first time, we have shown the benefit of using a DDP-4 inhibitor both with the ER structure seen under the electron microscope and with ER stress molecular markers.

Following the alleviation of ER stress in the liver, hepatic steatosis was also ameliorated in this study. NAFLD has been recognized as the most common liver disease in the western and eastern world, with an estimated prevalence of 20–30% [25]. The accumulation of fat, mainly triglycerides, within the hepatocytes is crucial in the development of NAFLD. One-third of total triglycerides might originate from de novo lipogenesis in the excessive accumulation of triglycerides in the liver, also as a result of lipid accumulation by reducing fatty acid oxidation [26]. Increasing research implies that ER stress is a central feature of peripheral insulin resistance and lipogenesis [27, 28]. In the stressed condition, the PERK-eIF2*α* and IRE1-xBP-1 pathways play potential roles in lipid accumulation in the liver. In one study, expression of hepatic xBP-1 led to decreased lipid synthesis [17]. Another study indicated that sustained dephosphorylation of eIF2*α* was associated with decreased lipogenesis and slight hepatic steatosis [18]. Among the studies on the mechanism of ER stress and hepatic steatosis, several observations have suggested that ER stress could trigger SREBP-1c cleavage and activation of lipogenesis [29–31]. As early as 2008, researchers proposed that liver steatosis could be prevented by a DDP-4 inhibitor des-fluoro-sitagliptin (DFS) due to a reduction in ER stress in the liver by GLP-1R signaling. But until now, there is no special study investigating whether vildagliptin can improve the high fat induced ER stress in the liver. In this study, ER stress in the liver was induced by high fat diet as indicated by increased protein expression of BiP, p-PERK/p-eIF2*α*, and p-IRE1*α*/XBP-1s, and along with ER stress in the liver, the intrahepatic lipid content increased, and mRNA expressions of genes associated with lipid synthesis, SREBP-1c, FAS, and ACC1 were all upregulated; in contrast, ER stress was reduced, mRNA expression of genes associated with lipogenesis was downregulated, and hepatic lipid deposition was ameliorated in mice treated with vildagliptin. Activated PPAR*α* is related to fatty acid metabolism; activated PPAR*γ* increases the mRNA level of ACC1 and FAS, coregulating the balance of hepatic fat metabolism. In our study, the mRNA expression levels of PPAR*α* and PPAR*γ* reduced after vildagliptin therapy. We think the decrease of PPAR*α* is related to the decrease of lipid content in the liver.

During ER stress, apoB100, which is the major apolipoprotein of VLDL, is decreased by inducing its cotranslational proteasomal degradation but without affecting its mRNA [32, 33]. VLDL synthesis and secretion decreased, and then export of hepatic triglyceride was impaired. In our study, serum triglycerides tended to decrease in HFD mice and increase in V-HFD mice. This suggested that vildagliptin promoted the secretion of triglycerides from hepatocytes into the blood, though we did not investigate the levels of apoB and VLDL. Increasing evidence indicates that chronic ER stress in the liver is a primary contributor to obesity-induced insulin resistance [34]. In our study, serum insulin tended to increase with the significant increase of fasting glucose in high fat diet mice, which tended to decrease with the decreased fasting glucose in mice treated with vildagliptin. The high chronic exposure to insulin can cause peripheral insulin resistance, which also induces steatosis by increased hepatic lipogenesis. Our work suggests that vildagliptin increased hepatic insulin sensitivity by preventing hepatic steatosis, and our results imply the possible involvement of the DDP-4 inhibitor vildagliptin in the complex interplay in the improvement of hepatic steatosis. The results of the current study demonstrate a novel therapeutic potential of vildagliptin for extrapancreatic effects.

In the past, DDP-4 inhibitors were thought to act through increasing the levels of GLP-1 and GIP by reducing their degradation. But growing evidence indicates a direct action for DDP-4 inhibitors, but it is not clear if this is through enzymatic or nonenzymatic actions. In a study, treated DPP4^−/−^ rats with normal or high fat diet displayed reduced hepatic triglycerides, accompanied by downregulation of lipogenesis enzymes and parallel upregulation of carnitine palmitoyltransferase-1, a key enzyme in fatty acid *β*-oxidation [35]. In vitro, a DPP-4 inhibitor significantly reduced LPS-induced vascular adhesion molecules, reduced inflammatory cytokine expression in human endothelial cells, and inhibited foam cell formation in monocytes [36]. In human mononuclear cells, a DPP-4 inhibitor reduced Toll-like receptor- (TLR-) 4 mediated production of proinflammatory cytokines [1]. Hwang et al. verified that a DPP-4 inhibitor inhibits ER stress-induced apoptosis and inflammation efficiently in cardiomyocytes [37]. The DPP-4 inhibitor gemigliptin significantly inhibited TNF*α*-induced mTOR phosphorylation, SREBP-1 cleavage, and lipid accumulation in HepG2 cells [38]. We treated HepG2 cells with PA, and the resulting ER stress was suppressed by vildagliptin. Meanwhile, we treated HepG2 cells with thapsigargin as a positive control and found that vildagliptin also alleviated the ER stress induced by thapsigargin. These results confirm the direct effect of vildagliptin on ER stress in HepG2 cells. Another DDP-4 inhibitor gemigliptin inhibited ER stress-induced apoptosis and inflammation effectively via PERK and IRE1*α* mediated pathways in H9c2 cardiomyocytes [37].

A limitation of the current study is the fact that we cannot investigate the levels of GLP-1 in serum and DDP-4 activity in the liver. Whether the direct effect of vildagliptin on alleviating ER stress is through enzymatic or nonenzymatic pathways needs further investigation.

In conclusion, vildagliptin can alleviate ER stress in the liver induced by a high fat diet following the decrease in triglycerides. Vildagliptin has a direct role in alleviating ER stress in vitro. Vildagliptin has potential as a potent agent in the treatment of fatty liver in addition to its wide use as a drug for lowering blood glucose.

## Figures and Tables

**Table 1:** Diet compositions.

	Normal control diet	High fat diet
Protein, g/100 g	20	17
Carbohydrate, g/100 g	58	49
Fat, g/100 g	6	21
Selenium, g/100 g	1.4 × 10^−5^	1.6 × 10^−5^
Cholesterol, g/100 g	0	2
Fatty acids, g/100 g		
C14:0	0.02	0.20
C16:0	0.97	5.62
C16:1	0.02	0.24
C18:0	0.21	2.05
C18:1	1.23	6.37
C18:2	2.57	3.49
C18:3	0.17	0.18
Total saturated	1.11	7.88
Total monounsaturated	1.22	6.78
Total polyunsaturated	2.93	3.67
Total kcal/g	3.4	4.1

**Table 2:** Sequences of the primers used for evaluation of the relative mRNA expression levels by real-time PCR.

Gene	Forward	Reverse
*β*-actin	ACCCCAGCCATGTACGTAGC	GTGTGGGTTACCCCGTCTC
SREBP-1c	GCGCTACCGGTCTTCTATCA	GGATGTAGTCGATGGCCTTG
FANS	GTCCTGGGAGGAATGTAAACAG	CGGATCACCTTCTTGAGAGC
ACC1	GCTTATTGATCAGTTATGTGGCC	CTGCAGGTTCTCAATGCAAA
PPAR*α*	AAGGGCTTCTTTCGGCGAAC	TGACCTTGTTCATGTTGAAGTTCTTCA
PPAR*γ*	GACCTGAAGCTCCAAGAATACCA	CCCACAGACTCGGCACTCA

**Table 3:** Blood biochemistry measurements in the three groups.

	CD group	HFD group	V-HFD group
Glucose (mmol/L)	5.76 ± 0.97	9.06 ± 1.69^*∗∗*^	7.46 ± 1.50^#^
Insulin (nIU/ml)	155.4 ± 33.0	222.1 ± 29.6^*∗*^	165.3 ± 38.2^#^
TG (mmol/L)	0.37 ± 0.04	0.33 ± 0.06	0.46 ± 0.13^#^
TC (mmol/L)	2.00 ± 0.37	3.93 ± 0.88^*∗∗*^	3.28 ± 1.11
LDL (mmol/L)	0.34 ± 0.10	0.70 ± 0.17^*∗∗*^	0.53 ± 0.13^#^
HDL (mmol/L)	1.45 ± 0.18	2.80 ± 0.73^*∗∗*^	2.41 ± 0.88
ALT (U/L)	50.6 ± 7.6	91.7 ± 32.0^*∗∗*^	65.5 ± 19.7^#^

TG = triglyceride, TC = total cholesterol, LDL = low density lipoprotein, HDL = high density lipoprotein, ALT = alanine aminotransferase. Data are mean ± SD *n* = 8–10. ^*∗*^*P* < 0.05 relative to CD group. ^*∗∗*^*P* < 0.01 relative to CD group, ^#^*P* < 0.05 relative to HFD group.
